# Investigation of the Release Rate of Biocide and Corrosion Resistance of Vinyl-, Acrylic-, and Epoxy-Based Antifouling Paints on Steel in Marine Infrastructures

**DOI:** 10.3390/polym15193948

**Published:** 2023-09-29

**Authors:** Adel Jalaie, Abdolah Afshaar, Seyed Borhan Mousavi, Mohammad Heidari

**Affiliations:** 1 Department of Materials Science and Engineering, Sharif University of Technology, Tehran 1458889694, Iran; 2 J. Mike Walker ‘66 Mechanical Engineering Department, Texas A&M University, College Station, TX 77843, USA; 3 Faculty of Chemical & Petroleum Engineering, University of Tabriz, Tabriz 5166616471, Iran

**Keywords:** antifouling (AF), Cu_2_O/ZnO biocide, polyaniline, vinyl paint, acrylic paint, epoxy paint

## Abstract

This study comprehensively assesses the release rate of biocides, corrosion effects related to antifouling, and the physical properties of different paint types. Tests were conducted to measure thickness, viscosity, hardness, bending, adhesion, gloss, impact resistance, abrasion resistance, scratch resistance, polarization, and salt spray. The paints evaluated include resin-based, acrylic-based, epoxy-based, and vinyl-based formulations. The study investigates the influence of biocide content, biocide particle size, and immersion time on release rate using a lab-scale setup. Results showed that acrylic-based paints had a higher biocide release rate due to faster hydrolysis, while smaller biocide particle sizes led to higher release rates in resin-based paints. Optimal total biocide contents were determined to be 30% for acrylic-based, 60% for epoxy-based, and 50% for vinyl-based paints. Antifouling corrosion analysis demonstrated that sample with an optimal release rate effectively prevent algae growth and fouling. Acrylic-based paint with 30 wt.% biocide content exhibited superior adhesion with a dolly separation force of 4.12 MPa. Evaluating the impact of synthesized polyaniline on 30 wt.% epoxy-based paint, a sample coated with 10 wt.% polyaniline represented a low corrosion rate of 0.35 µm/year and a high impedance value of approximately 37,000 Ohm·cm^−2^.

## 1. Introduction

Accumulation and adherence of marine organisms on submerged surfaces, known as biofouling, can cause drastic harmful influences on manufactured marine infrastructure during its service time, leading to greater fuel consumption, arduous hull cleaning processes, expedited corrosion, and a need for more maintenance [1,2,3,4]. Hence, developing practical and durable antifouling (AF) approaches is vital to preserving marine infrastructure from biofouling organisms. To overcome the mentioned drawbacks, antifouling paints are usually used to constrain the organisms’ growth on the ship’s body [5]. Recently, while numerous investigations have experimented with utilizing biocide-free antifouling methods, namely bio-inspired coatings, fouling-resistant coatings, and fouling-release coatings, biocides have been mostly used as the principal effective components in commercial antifouling coatings [6,7,8,9]. Microbial biofilms have been observed to perform an essential part in interfering with the establishment of invertebrate larvae and microorganisms from fungi, which is the primary form of fouling. Recent research has proposed that the relationship between microbial biofilms and the growth of consequent macrofouling areas could be notable [10,11,12]. Tributyltin self-polishing copolymer (TBT-SPC) paints have been used widely for more than two decades due to their remarkable antifouling features; however, tributyltin-based antifouling coatings have been forbidden due to environmental issues. Since the complete prohibition of the usage of tributyltin (TBT) as a biocide in the marine ecosystem, copper oxide (Cu_2_O) has attracted attention as an antifouling biocide in marine paints due to its substantial characteristics such as durability, performance, and effectiveness; in addition, antifouling paints including copper oxide can provide sufficient fouling control to a great number of steels [13,14,15,16]. Lately, considerably conducting polymers (ICPs) such as polyaniline (PANI) and polypyrrole (PPy) and their derivatives have been investigated due to their outstanding features with regard to reversible electrochemical performance, electrical conductivity, optical and electrical traits, etc. [17,18]. In addition, these materials have been used extensively in industrial applications, namely batteries, electronic devices, and sensors, and as anticorrosive additives due to their promising features such as excellent mechanical and physicochemical attributes, low costs, and safety [19,20,21,22]. Since a thin film of conducting polymers could preserve the substrate only for a short period, organic coatings and conducting polymers are usually combined to achieve effective and durable corrosion resistance [23,24,25]. Numerous studies have focused on examining the corrosive characteristics of PANI coatings, proving that with the addition of nanoparticles (NPs) to the coatings, better anticorrosive properties will be gained due to the formation of a stable passive film on the surface of the substrate. These nanoparticles include Fe_2_O_3_ [26,27,28], CNT, ZnO, ZrO_2_ [29], activated carbon [30,31], and TiO_2_ [32,33,34,35,36,37,38,39]. Zinc oxide (ZnO), an inorganic material, has been widely used in many applications such as photocatalysis [40], optics, and heat transfer [41] and especially for antibacterial purposes [42,43].

Yong et al. [42] utilized ZnO NPs in an alkyd resin to prepare a biocide antifouling paint. The results confirmed that ZnO NPs could potentially coat infrastructure in marine industries. Al-Belushi et al. [44] first prepared ZnO nanoparticles to fabricate chitosan (CHT)-ZnO NPs coatings at two different chitosan solutions, CHT 1% and CHT 2%. Then, they assessed the antifouling features of the prepared coatings using Gram-negative and- positive bacteria. The outcomes revealed that the prepared specimens had higher antifouling features compared with the pure nano-ZnO rods or CHT coatings than those of commercially available paints in the dark and under light irradiation. They further asserted that the fabricated coatings had better efficacy in the removal of Gram-negative bacteria rather than Gram-positive ones. Rajan et al. [45] conducted a study to investigate epoxy-based paint’s antifouling and anticorrosive characteristics by applying the disc diffusion method. The results showed that the investigated properties of the measured paint could maintain antifouling and anticorrosive characteristics in natural seawater for about six months.

Moreover, the effectiveness and durability of the coating can be improved by raising the G. edulis extract contents. Ni et al. [46] evaluated the preparation, antifouling, and self-polishing attributes of the multifunctional resins by hindering algae addition analysis, anti-protein adsorption, and antifouling test. They concluded that the acrylic metal salt resins, including the indole derivative, had substantial antifouling and self-polishing attributes compared to pure acrylic metal salt resins. Adeleye et al. [47] studied the chemical state of copper particles in water and the effect of drying time, surface type, and water salinity on the release rate of different copper particles. They asserted that the release rate of copper increased with water salinity. They further stated that drying paints for longer than what is suggested by the manufacturer will cause the additional release of biocides into water. Palanichamy et al. [48] conducted a study to evaluate the antifouling behavior of five different marine bacteriocins using epoxy-based paint. The outcomes demonstrated that the prepared coatings could maintain antifouling behavior for three months in seawater, and the life expectancy could be improved as biocide content increased. Quan et al. [49] conducted a study to evaluate the antifouling, antibacterial, and anticorrosion behavior of epoxy coatings containing prepared poly(m-aminophenol) (PmAP) at four different concentrations. The antibacterial characteristics were measured via the plate count determination method, showing that sufficient cationic moieties and phenolic hydroxyl groups available in PmAP had effective antibacterial activity toward B. subtilis and E. coli. It was further asserted that the antifouling feature of the prepared coatings increased with the increase in concentration; thus, the coatings were preserved from failure affected by microbes. Quan et al. [50] investigated the antifouling and antibacterial execution of a binary epoxy coating comprising bromine-benzyl-disubstituted polyaniline (BBP). The comprehensive analyses confirmed that the prepared samples had better antifouling execution than the normal PANI coating, and the optimal concentration was 5%. Moreover, it was found that the antibacterial properties of the coatings were enhanced with the addition of BBP nanoparticles. Tonelli et al. [51] analyzed the efficacy of utilizing halloysite clay nanotubes (HNTs), which are nanocontainers, on the antifouling behaviors of epoxy coatings, proving that the measured properties of the coatings were enhanced with the incorporation of used nano-containers. The antifouling capability of biodegradable polyurethane was examined by Ma et al. [52]. They reported that the composition of the polyurethane could control the antibiofouling capacity.

Based on the performed literature review, thorough examinations have been carried out on the release rate of biocides and the corrosion rate of resin-based paints, while limited considerations have been paid to the release rate of disparate biocides and the corrosion rate of different resin-based paints using wide weight ratio range of additives to acquire the optimum concentrations. Moreover, a novel bimetallic biocide containing two different biocides, Cu_2_O and ZnO, was utilized to prepare all resin-based paints. In addition, a novel lab-scale setup using artificial seawater was designed and built with a high similarity in composition and concentration to realistic seawater to measure the release rate.

In this investigation, we aimed to consider all affecting parameters of the antifouling properties of different biocides, environmental issues, and physical features of the various coatings. Moreover, all essentially required industrial aspects were comprehensively considered to assess the industrialization facilities, confirming that the mentioned coatings could remarkably ease industrialized capabilities, which, to the best of our knowledge, has not been studied so far.

In this study, the biocidal release rate should be examined to achieve a suitable formulation for antifouling paint, which is the most important component in the paint formulation to combat growth fouling. For this reason, first, the basic formulation of paints was extracted from the articles, and then by changing the effective parameters, such as the type of resin, the content of the biocide in the paint, and the grain size of the pigments on the release rate, the most appropriate formulation was selected. Mechanical, physical, corrosion resistance, and paint quality tests were performed on St37 steel substrate for samples at each optimal formulation. In order to guarantee the consistency and replicability of outcomes, every experiment was performed a minimum of three times.

## 2. Experimental Materials and Methods

### 2.1. Materials

Commercial Cu_2_O and ZnO were purchased from Sigma-Aldrich and Merck, respectively. To formulate antifouling paints, acrylic resin No. 2378, EPON 828 resin, and Mowital B 30 H polyvinyl butyral (PVB) were obtained from Taak Resin Kaveh Chemical Co. (Tehran, Iran), Shell Chemical Co. (Deer Park, TX, USA), and Kuraray (Merrillville, IN, USA), respectively. Zinc phosphate epoxy No. 15300 and polyamine epoxy No. 45180 to be used as primers were obtained from Bajak Paint Co. (Tehran, Iran). To prepare the specimens, St-37 steel was used as the base metal with a thickness of 2 mm. The chemical composition of the used steel is listed in Table 1.

After preparing the samples, abrasive blasting was conducted to increase the surface roughness and adhesion of the coating and remove possible oxide layers on the surface. Then, the specimens were cleaned using acetone to remove surface contaminants such as grease. To examine the biocide release rate, glass plates with dimensions of 10 × 15 cm^2^ were used.

### 2.2. Paints Preparation

To prepare the acrylic-based paint, first xylene and *n*-butanol were mixed, at an appropriate ratio of each (1:1), using a stirrer at 2000 rpm for 30 min, then acrylic resin powder with other additives was added in the ratio listed in Table 2. The mixture was stirred at 2000 rpm for 1 h; last, Cu_2_O and ZnO were added to the mixture at different contents. The particle size was measured using a grindometer which was set to 50 µm. By adding glass beads at a ratio of 1:2 to the prepared sample and mixing for about 30 min, the particle size decreased to 20 µm. The paint particles were separated from glass beads using a metal mesh [53].

To prepare an epoxy-based paint, liquid resin and the additives listed in Table 2 as well as Cu_2_O and ZnO biocides, were mixed at 2000 rpm for 1 hr. The glass beads were utilized to decrease the particle size. One hour prior to the application, the paint was mixed with a Triethylenetetramine (TETA)-based amine hardener (Triethylenetetramine, Aradur^®^ HY951, Huntsman Corporation, Mumbai, India) at a ratio of 100 to 15.

Rosin particles must be dissolved to be used in surface coating. Thus, the rosin particles (CAS No: 63449-39-8, Rosin Factory, Clifton, NJ, USA) were powdered well using a mortar. The rosin powders were mixed with xylene at a ratio of 1:2. Mowital B 30 H polyvinyl butyral (PVB) was dissolved into cyclohexane using a stirrer at 2000 rpm for about 30 min. The listed additives in Table 2 and biocides were also mixed at the same speed and time. Eventually, the prepared rosin was mixed with the PVB mixture with a ratio of 1:2 for 30 min. Particle size was decreased using the glass beads.

### 2.3. Curing Time

The paints were applied to the plates using a 60 μm applicator at 22–25 °C, and the curing time was determined based on the ISIRI6456 standard [54,55]. All tests were carried out three times to minimize error and ensure the repeatability of data.

### 2.4. Biocide Release Rate Measurement

A laboratory setup was sketched and built based on the ASTM D6442-06 [56,57]. A layer of the prepared paints with a thickness of 120 μm was applied to the glass plates to an area of 10 × 15 ${cm}^{2}$ using an applicator. The laboratory system consisted of a container that was filled with artificial seawater. The artificial seawater was prepared according to the ASTM D1141 standard [58,59], and its specifications are listed in Table S1. The temperature and pH of the water were maintained at 23 ± 2 °C and 7.9–8.1, respectively, and they were monitored continuously during the tests. The size ratio of the painted plates in the water and the total amount of water in each chamber were maintained at between approximately 1:3–1:6. A 1000 cm^3^ quantity of artificial water was poured into each chamber. To create dynamic and wave-like seawater conditions, blowing was carried out in each chamber by channeling the air pump, which caused turbulence in the water. The outlet air flow rate for each chamber was determined via a flowmeter and kept at 60 ± 5 mL/min. Figures S1 and S2 present the built laboratory setup and its corresponding schematic, respectively.

Cu concentration was evaluated at 5-, 10- and 15-day intervals by sampling 20 cm^3^ of water in each chamber and applying atomic absorption spectroscopy (AAS). The reason for choosing 15 days is that the release rate was investigated when the water had completely penetrated the paint layer, breaking and reforming the penetration barriers of the paint. It was demonstrated that penetration and biocidal dissolution occurred in 7 days [60,61]. Although the water evaporated during the tests, the initial water level was maintained by adding distilled water to the chambers. The release rate was calculated by Equation (1) [56].

(1) $$R=\frac{CVD}{AT}$$

where R, C, V, D, A, and T are the release rate of biocide ($\frac{\mu g}{{cm}^{2}.day}$), the content of biocide (($\frac{\mu g}{L}$), the volume of seawater in the measuring container (L), the number of hours per day (24), the surface area of the paint film (${cm}^{2}$), and the time the flat panel was immersed in the measuring container (h), respectively.

If the calculated release rate is $\geq 15\frac{\mu g}{{cm}^{2}.day}$, it means that the paint has antifouling activity, but for $R<15\frac{\mu g}{{cm}^{2}.day}$, the antifouling performance of the paint is not satisfying [62].

### 2.5. Antifouling Resistance Analysis

A laboratory setup was built to examine the antifouling features of the samples. The setup consisted of two glass tanks under the radiation of artificial light with UV lamps with an intensity of about 90 lux that provided necessary light to the algae at night and accelerated their growth. An air and CO_2_ gas blower pump were also provided in the setup. A photo of the setup is presented in Figure S3. The water temperature was maintained at 27–30 °C. The chemical composition of the water used in the tanks is listed in Table S2. The samples with satisfying biocide release rates were considered in this test, and the algae formation process was analyzed. The samples contained substrates, zinc phosphate epoxy No. 15300, polyamine epoxy No. 45180, and antifouling paint. A thickness of 150 μm of each layer was applied to the steel plate, and the painted samples were compared to the pure ones. Each sample was kept in the system for 3 months.

### 2.6. Thickness Measurement

The thickness of the samples was studied using the PosiTector 6000 (DeFelsko, The Woodlands, TX, USA) instrument based on ASTM D1186 [63,64]. The tests were conducted at 5 points for each sample, and the mean value was reported.

### 2.7. Viscosity Measurement

Since the viscosity of paints plays an influential role in their properties, to remove this parameter, the tests had to be conducted at fixed viscosity; thus, the viscosity of all samples was kept at 110 Krebs by employing a Brookfield (Ametek, Middleborough, MA, USA) viscometer at 25 °C temperature based on ASTM D562 [65,66].

### 2.8. Hardness Test

To measure the hardness of the samples, the pencil hardness and König pendulum hardness tests were performed according to ASTM D3363 and D4366 standards [67,68], respectively [69,70]. The pencil hardness and König pendulum hardness tests were conducted employing Wolff-Wilborn (BYK, Germany) and byko-swing König (BYK, Geretsried, Germany) instruments, respectively. The samples were prepared by applying a layer of the formulated paints at a thickness of 120 μm.

### 2.9. Bending Test

The analyses were performed by spraying a layer of the prepared paints at a thickness of 120 μm on the 10 × 15 ${cm}^{2}$ plates after 1 week according to ASTM D522 standard [69,71]. The tests were conducted using a Mandrel bender machine (Ercolina, Davenport, IA, USA).

### 2.10. Paint Adhesion Test

The adhesion tests were performed using a Pull-off adhesion tester (DeFelsko PosiTest AT, The Woodlands, TX, USA) according to ASTM D4541 standard [72] for a layer of the prepared paints with a thickness of 120 μm [73].

### 2.11. Gloss Test

The gloss tests were conducted for a layer of the prepared paints with a thickness of 120 μm at 60° angle utilizing a gloss meter apparatus (3NH NHG60, Ramsey, NJ, USA) based on ASTM D523 standard [74,75].

### 2.12. Impact Resistance Test

The impact resistance tests were conducted utilizing an impact resistance machine (HD-A520, Haida international, Dongguan, China) based on the ASTM D1794 standard [76]. In this test, the deformation of the coating was evaluated with a 2-pound ball released from different heights. Three samples were prepared for each test.

### 2.13. Abrasion Resistance Test

This test was conducted to measure the life of the paints against abrasion. A layer of the prepared paints with a thickness of 200 μm was applied to an area of the plate with dimensions of 100 × 100 × 1 ${mm}^{3}$, having an 8 mm hole in its center. A 1 mg quantity of paint was placed on the machine’s rotational surface, the abrasive wheels were placed tangentially to the sample’s surface, and a 250 g loading bar was applied to each. A suction device was installed close to the abrasion site, at a distance of 1 to 2 mm from the sample’s surface, to remove dust caused by abrasion to the device from the test site. The mass loss of the samples was measured after 200 cycles of wear compared with the initial weight. The tests were conducted using an abrasion resistance test machine (GT-C14A, Nanjing, China) according to ASTM D4060 standard [77,78].

### 2.14. Scratch Resistance Test

Scratch resistance tests of the samples were performed at different scratch forces, 5−30 N, employing a scratch resistance tester (SMT-500, Rtec, San Jose, CA, USA) according to ASTM D7027 standard [79,80].

### 2.15. Polarization Test

To study the corrosion behavior of the prepared resins and the effect of polyaniline as an additive in increasing the corrosion resistance of antifouling paints, the polarization curves by Autolab in the range of −0.6 to 0.6 V relative to the open circle potential with a sweeping rate of 1 $\frac{mV}{s}$ in the formulated 3.5% NaCl solution was used. Prior to the tests, the samples were placed in the solution for 10 min to achieve equilibrium conditions. According to ASTM G102-89 standard [81], the extrapolation method was used to calculate samples’ corrosion rate from polarization curves [82].

### 2.16. Salt Spray Test

Salt spray tests were performed to study the corrosion behavior of the samples according to ASTM B117 standard [83,84]. The samples were placed inside the chamber so that they were at an angle of about 15 to 30 degrees to the vertical axis and had no contact with each other. To speed up the conclusion time, scratches were applied to the samples up to the depth of the base metal surface and along the diameter. The dimensions of the salt spray test samples in the study were 2 × 5 ${cm}^{2}$ and included three coats of primer, middle paint, and antifouling paint. The duration of the test was 240 h. To prevent the penetration of corrosion from the back and edges of the sample, the back of all the samples was protected with varnish and adhesive tape.

### 2.17. Polyaniline Synthesis

In this study, polyaniline was synthesized, and its antifouling property and efficacy against corrosion were investigated. A 20 g (0.1 mol) quantity of ammonium persulfate was dissolved as oxidizer and initiator of the polymerization reaction in 100 cm^3^ of an aqueous 1 M perchlorate acid solution. A 0.1 mol quantity of double-distilled aniline (10 g) in 100 cm^3^ of an aqueous 1 M perchlorate acid solution was dissolved. The initiator solution was added dropwise to the monomer solution, and the reaction temperature was stabilized at 0 °C. The primer solution was added to the polymerization container within 1 h, and the reaction mixture was stirred with a magnetic stirrer. After this time, the contents of the container, which contained a blackish-green precipitate, were filtered through a Buchner funnel. The resulting polymer was washed under filtration with methanol. The polymer was then washed with distilled water to remove residual methanol and dried in an oven for one day. After the synthesis of polyaniline powder, it was mixed with dimethylformamide as a solvent in a ratio of 1:1. Previous studies added different amounts of polyaniline/acid to the paint [85,86]; however, the best performance in increasing the corrosion resistance and antifouling qualities has been obtained in the range of 10–20 wt.% [87]. Therefore, 30 wt.% epoxy-based antifouling paints were prepared with the addition of 10, 15, 20, or 30 wt.% of polyaniline/acid. The prepared antifouling paints were applied to the surface of the steel with a thickness of 150 µm, and the results of the polarization and impedance tests were compared with the sample without polyaniline. Then, the optimal content of polyaniline added to the paint was determined.

## 3. Results and Discussion

### 3.1. Biocide Release Rate Measurement

#### 3.1.1. Effect of Resin and Immersion Time on the Release Rate

Release rates of Cu_2_O and ZnO biocides for paints with a grain size of 20 µm at 5-, 10-, and 15-day intervals are listed in Table 3. Based on the release rates of Cu_2_O and ZnO biocides, it can be seen that in the case of paints in which acrylic resin was used in their preparation, the release rate was higher than that of the other two resins due to its polishing property. Binders and polymer chains can be hydrolyzed in acrylic copolymers. Because of the hydrolysis of their ester groups when in contact with water, acrylic resins weaken the bond between the binder and the biocides, releasing them more easily [88]. This reaction occurs in the highest layer in the presence of Cu_2_O and ZnO biocides, which have partial solubility in seawater. Below this top layer, which is the paint’s surface, the unhydrolyzed polymer remains completely hydrophobic until the bottom layer is exposed to seawater due to the dissolution of the top hydrolyzed layer. Due to the hydrolysis of the acrylic paint layer, the Cu_2_O and ZnO biocides are also separated from the polymer and are available to act as antifouling agents. Once the hydrolyzed polymer is removed from the surface, the lower surfaces of the layer encounter water, and hydrolysis continues at the new binder surface. The biocides obtained from layer hydrolysis increase the release rate, resulting in higher release rates of Cu_2_O and ZnO in paints with higher contents than the other two resins. Due to its intrinsic physical properties, the biocide release rates of epoxy resin paint were lower than the obtained values for acrylic and vinyl resin paints. Epoxy resin is used for insoluble matrices in antifouling paints. Since epoxy resin has a high crosslinking density, water can only fill the created pores by the dissolution of biocides and pigments on the paint surface; thus, seawater penetration paths to wash biocides out of the paint are low. Over time, the seawater gradually dissolves more biocides, expanding the infiltration layer. When a certain amount of biocide is removed from the surface of the paint layer, the movement of seawater easily erodes the brittle polymer chain, and the layer with less soluble biocides is exposed to seawater, which causes the lower biocide release rate of epoxy-based paints. At high contents of biocides, better antifouling performance was achieved.

Vinyl/rosin-based paints had release rates that were lower than acrylic-based paints but higher than epoxy ones. This behavior could be due to the simultaneous presence of two types of resins with different properties in these paints. Vinyl resins have a high degree of polymerization and are resistant to seawater, which causes the pigments to be locked in this type of resin, and the porosity is greatly reduced. On the other hand, rosin is a natural substance obtained from pine gum, which is chemically very sensitive to alkaline conditions. If the solubility rate is not controlled in the alkaline environment, the seawater dissolves the paint rapidly, and the paint disappears. The antifouling coating quickly loses its effectiveness. For this purpose, hydrophobic or non-polar compounds should be used to control the rate of paint solubility. Mixing rosin with hydrophobic vinyl hydrolyzes the binder and increases the biocide release rate. According to the results of the release rate test, it can be said that in general, the release rate of biocides depends on the rate of hydrolysis of the copolymers from which the paint is made. Therefore, acrylic copolymers have a higher hydrolysis rate than epoxy, and vinyl copolymers have a higher biocide release rate. In epoxy copolymers, the rate of hydrolysis and release decreases due to the increase in the side chain length of the monomers. In addition, with an increase in the chain length and the spatial barrier of the lateral branches, the polarity of the resin decreases, and its hydrophobicity increases; moreover, the rate of water penetration and water contact with the resin decreases. In vinyl resin, hydrolysis and release rates increased with the rise of the reactive rosin group.

Table 3 shows that the release rates after 5 days were higher than those after 10 and 15 days for all paints. In the first days of the measurement, a sharp increase in release rates of biocides was seen for all prepared resins due to the high content gradient created between the paint and the aqueous environment because of the high biocide content in the paint’s surface layer. The biocides were transferred out of the coating by forming a biocide content gradient, and a relative density was created in the boundary layer adjacent to the coating. The interesting point here is that after the first few days, the release rate of all prepared resins reached a steady state, and no remarkable change was seen. In the case of acrylic resin, due to the nature of the binder, which could be hydrolyzed, it always caused a new layer to be replaced as the biocides were eliminated in each layer, and therefore, the release rate decreased after the fifth day.

In the case of epoxy and vinyl/rosin resins, as mentioned, in the first five days, due to the high content of biocides on the paint surface, the release rate was high. However, after that, the release rate decreased, and the reason for this could be related to the formation of penetration barriers in the resin. Since epoxy resin is insoluble, water must penetrate via the pores created by the dissolution of the initial biocides on the surface into the paint. Over time, the penetration path became longer as the surface biocides were eliminated. After a certain time, the expansion rate of the depth of the pores due to the dissolution of pigments and the penetration of ions from the penetration layer was less than the biocide release rate and created a barrier against the penetration and release of biocides. After a thickness of 10 to 20 µm, these created barriers in the paint, causing the release rate to remain relatively constant until the end of the paint life.

#### 3.1.2. Effect of the Content of the Biocides on the Release Rate

**Figure 1:**
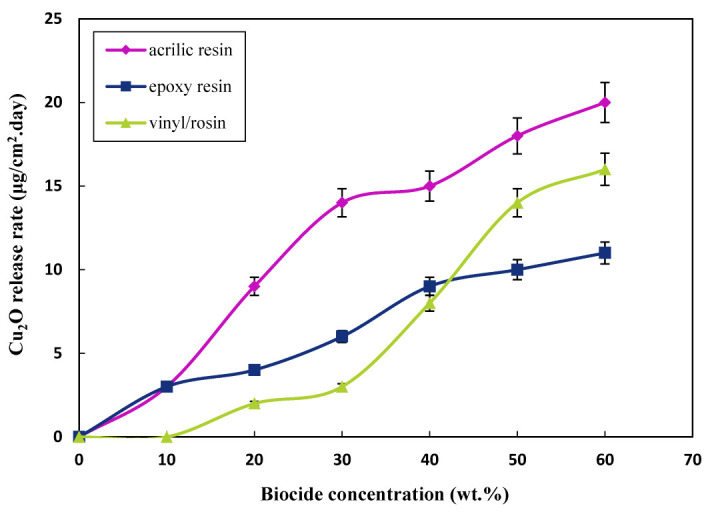
Variation of Cu_2_O release rate at different contents in steady-state condition after 5 days.

**Figure 2:**
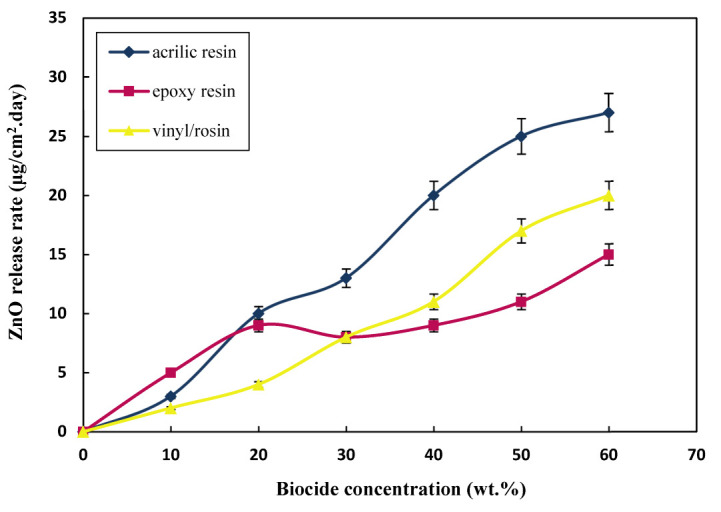
Variation of ZnO release rate at different contents in steady-state condition after 5 days.

Figure 1 and Figure 2 show the relationship between the content of the biocides and the mean release rate of Cu_2_O and ZnO in a steady-state condition; steady-state refers to the biocide release rate after five days, when the release rate reached an almost constant rate for all prepared resins. Considering Figure 1 and Figure 2 and Table 4, it can be seen that the release rate increased linearly with the increase of the content of biocides; however, at higher contents, the release rate almost reached a fixed state, and no remarkable change was seen. This is highlighted more for acrylic resin, since the amount of biocide available in each layer of paint remained fixed after 40 wt.% content of biocides. The mechanism of this type of paint is the simultaneous dissolution of the paint layer and biocides; thus, increasing the content of biocides did not affect the available amount of biocides in the most superficial layer of the dissolving paint. In the case of epoxy and vinyl paints, the release rate was fixed at higher contents since, in this type of insoluble resin, the particles are in physical contact with each other, and as the particle leaves the paint and enters seawater, a path for another particle to be exposed to the aquatic environment is created, so the release rate at high contents continues to increase slightly [60].

Another point to consider here is the comparison of the release rates of Cu_2_O and ZnO. The main biocide in antifouling paint is Cu_2_O, and ZnO is used as an additional biocide in paint. Additionally, due to the high release rate of ZnO compared to Cu_2_O, the concentration of ZnO in the formulation was half that of the Cu_2_O (1:2). This can be well demonstrated by comparing the release rates of the two biocides. However, when the concentration of ZnO in the formulation was half that of Cu_2_O, the release rates of the two were almost close in all cases. In addition to the higher partial dissolution of ZnO relative to Cu_2_O in seawater, the lower density of ZnO also helps it to exit faster and penetrate more easily [89].

Moreover, due to the lower dissolution rate of Cu_2_O, the dissolution of Cu_2_O biocides controls the release rate kinetics. However, it is worth stating that with the faster dissolution of ZnO, pores and penetration paths for water entry and Cu_2_O penetration increase. Accordingly, the Cu_2_O release rate increased, especially in insoluble epoxy resin-based paints. Another equally important point is that, since ZnO biocides are lighter than Cu_2_O biocides, after spraying the paint, its concentration on the surface of the paint will be higher than that of the inner layers; after the dissolution of ZnO on the surface, the water penetration path will be deeper.

ASTM D6442-06 states that the release rate of paint should be in the range of 10–15 ($\frac{\mu g}{{cm}^{2}.day}$) to have a good antifouling property. If the paint has a higher release rate than 15 ($\frac{\mu g}{{cm}^{2}.day}$), it poses a danger to other organisms in the marine environment, and using this type of paint is inappropriate. Accordingly, Table 4 lists the optimal content of biocides showing antifouling activity.

#### 3.1.3. Effect of Biocide Particle Size on the Release Rate

To investigate the effect of the biocide particle size on the release rate, two different sizes were considered, 20 µm and 50 µm, and the tests were conducted at three intervals, as before. Table 5 lists the computed release rates for three prepared resins at different contents, test duration, and particle sizes. Based on the release rate values listed in Table 5, lower release rates were acquired for 50 µm particles in comparison with 20 µm ones for all samples, since the smaller biocide particles had a better chance of penetrating rather than the larger ones. Furthermore, as surface-to-volume ratio increased, after dissolution, the 20 µm particles created more pores in the paint surface for water to penetrate, which in turn increased the release rate. Smaller biocide particles also had uniform distribution, leading to a higher release rate. However, according to the results, with increasing the biocide particle size, the release rate reduction for epoxy and vinyl/rosin resins was greater than that of acrylic resins, which confirms that these resins had a penetration release mechanism.

### 3.2. Effect of Biocide Release Rate on the Paint Thickness

**Figure 3:**
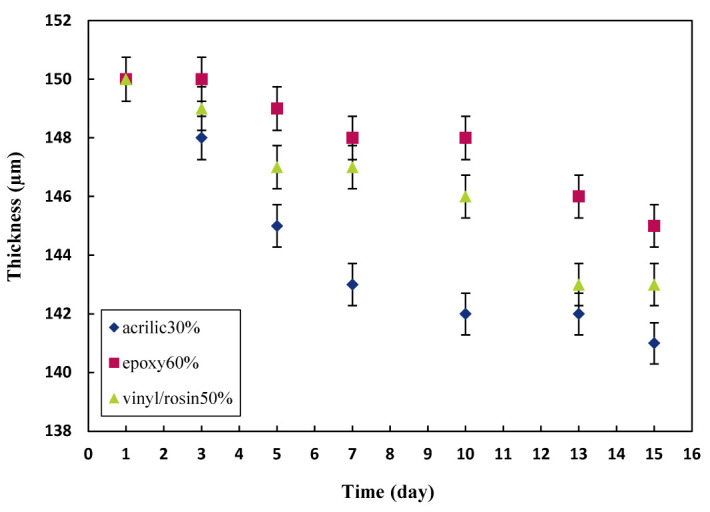
Thickness of the samples as a function of time at optimal content.

To investigate the effect of biocide release rate on the thickness of the paint layer, first, steel samples were coated with two paint substrates with a total thickness of 150 µm. Then, a layer of antifouling paint with the optimal content of each prepared resin was applied (Table 5). The backs of the samples were covered entirely with a chlorinated rubber resin-based swimming pool coating (Pars Pamchal Co.) to prevent corrosion and errors in the release rate calculations. The release rate experiments were carried out, and the thickness of the paints was measured for 15 days (Figure 3). As shown in Figure 3, the largest reduction in thickness over time was related to acrylic resin, which was due to the hydrolyzable nature of the resin.

### 3.3. Antifouling Resistance Analysis

**Figure 4:**
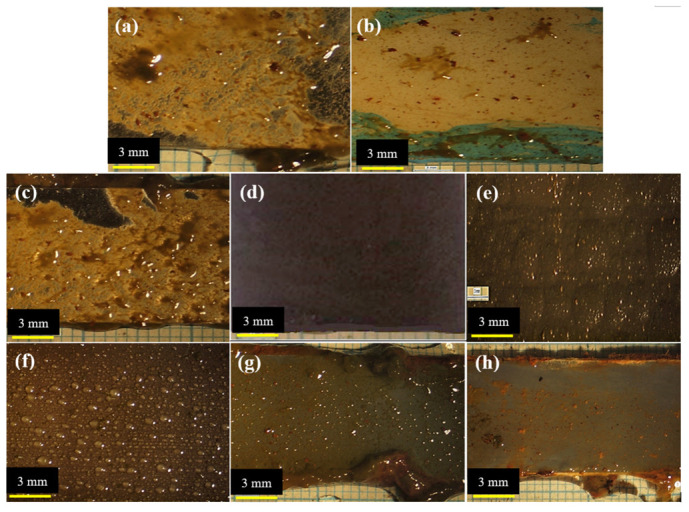
Real photos after 3 months of sample (**a**) without antifouling coating, (**b**) with substrate and without antifouling coating, (**c**) 20 wt.% epoxy-based antifouling coating, (**d**) 60 wt.% epoxy-based antifouling coating, (**e**) 10 wt.% acrylic-based antifouling coating, (**f**) 30 wt.% acrylic-based antifouling coating, (**g**) 20 wt.% vinyl/rosin-based antifouling coating, (**h**) 50 wt.% vinyl/rosin-based antifouling coating.

The antifouling resistance analyses were performed considering the optimal content of each resin. Some samples with a release rate lower than 10 ($\frac{\mu g}{{cm}^{2}.day}$) were randomly selected to investigate the correct functioning of the designed setup. The results were shown as a real photo after 3 months Figure 4. Sample (a) was without antifouling coating; as a result, the effect of fouling was seen after 3 months on the surface of the sample. Algae and fouling larvae were observed on the surface of sample (b). The release rate of sample (c) was less than the amount required to deal with fouling growth based on the standard. In practice, however, it could not resist the growth of fouling agents. The release rate in sample (d) was optimal and resisted fouling growth, so no effect from algae or fouling was observed after 3 months. Although the release rate in sample (e) was lower than the counteracting fouling growth, the rate of fouling formation was not high. The brown particles present were related to the corrosion products of the substrate steel. The release rate in the sample (f) was such that it could resist fouling growth. The release rate of the sample (g) was less than the amount required to withstand the growth of fouling mentioned in the standard and practically could not resist fouling growth. Sample (h) had an optimal release rate, and no algae formed, but due to corrosion products on the edge of the sheet, brown particles were observed on the paint surface. Corrosion kinetics also intensified due to the lack of fouling formation.

### 3.4. Curing Time

The curing time for acrylic-based and vinyl/rosin-based paints was in the range of 25–30 min, and no fingerprint effects were observed after this time. Epoxy-based paints had a longer curing time than the other prepared samples, and the samples were dried after 8 h, but a fingerprint effect was observed. One of the important parameters affecting the initial curing time of a coating is the type of solvent in it. In the case of vinyl and acrylic resins, the curing time was almost the same for both due to the similar evaporation time of the solvent, regardless of the nature of their chemical structure. On the other hand, the curing time of epoxy resins was longer due to the chemical reaction between the resin and the hardener. It has been previously stated [60] that epoxy-based paint’s curing time is when the polymerization process is completed and the paint is at maximum hardness. In this study, the epoxy-based paints were completely dried after one week of application.

### 3.5. Hardness

The obtained results of the pencil hardness and König pendulum hardness tests at optimal contents of biocides are listed in Table 6. The graphite pencil hardness range is 8−1B, HB, F, and 1−6H, of which 6H was the hardest and 8B was the least hard. Based on the data listed in Table 6, it can be seen that the epoxy resin was the hardest, and the acrylic resin was the least hard of all. In the pencil hardness test, the sample must have a 2B degree of hardness, and in the König pendulum hardness test, the oscillation time must be >60 s to meet the standards. Variation of hardness depends on the type and nature of the resin. As the biocide content increased, the hardness degree of the paints increased, since the presence of a mineral compound in an organic resin increases the hardness of that resin. By comparing the obtained results of the release rate and hardness tests, it can be concluded that lower hardness was seen in the acrylic resin, which had a higher release rate at the optimal content of biocides. Therefore, the harder the resin, the lower the release rate, and in hard resins, such as epoxy, with high contents of biocides, optimal release can be achieved. The low release rate in harder resins is also due to more complex networks and higher crosslinking density, which increases the hardness of these resins and makes biocide release difficult. Furthermore, acrylic monomers involved in the polymerization process of acrylic resins, including ethyl acrylate, ethylhexyl acrylate, and acrylic acid, have low T_g_ temperature, which makes them less hard than the other two resins [90,91].

### 3.6. Bending Test

**Figure 5:**
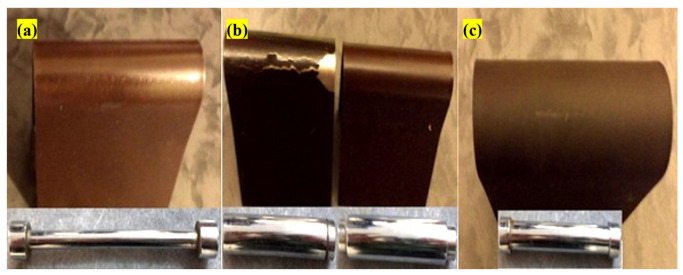
Photos of (**a**) acrylic resin, (**b**) epoxy resin, (**c**) vinyl/rosin resin after bending test.

ASTM D522 states that after bending painted specimens around cylinders with a maximum diameter of 6 mm, if the effect of destruction, cracking, and separation is not seen in the paint, as the paint’s flexibility is very high and desirable. If bending around cylinders with a diameter of 6 to 10 mm does not cause rupture or cracking, its flexibility is acceptable; otherwise, the paint will be poor in terms of flexibility. It is worth stating that the smaller the diameter of the cylinder or mandrel, the more intense the bending of the paint. Figure 5 presents photos of the prepared antifouling paints after the bending test. In epoxy resin-based paints, a 6 mm-diameter rupture was created due to bending around the mandrel. The paint was not damaged at a cylinder diameter of 8 mm, which is classified as suitable flexibility. The reason for this phenomenon is the epoxy resin’s high molecular weight. However, in the acrylic and vinyl/rosin-based paints, no bending effects were seen after bending around mandrels with a diameter of 3 and 5 mm, respectively, so they can be considered high-flexibility paints. The flexibility of the acrylic resin was due to its low molecular weight and the lower content of biocides in the optimal formulation of this resin.

### 3.7. Paint Adhesion Test

**Figure 6:**
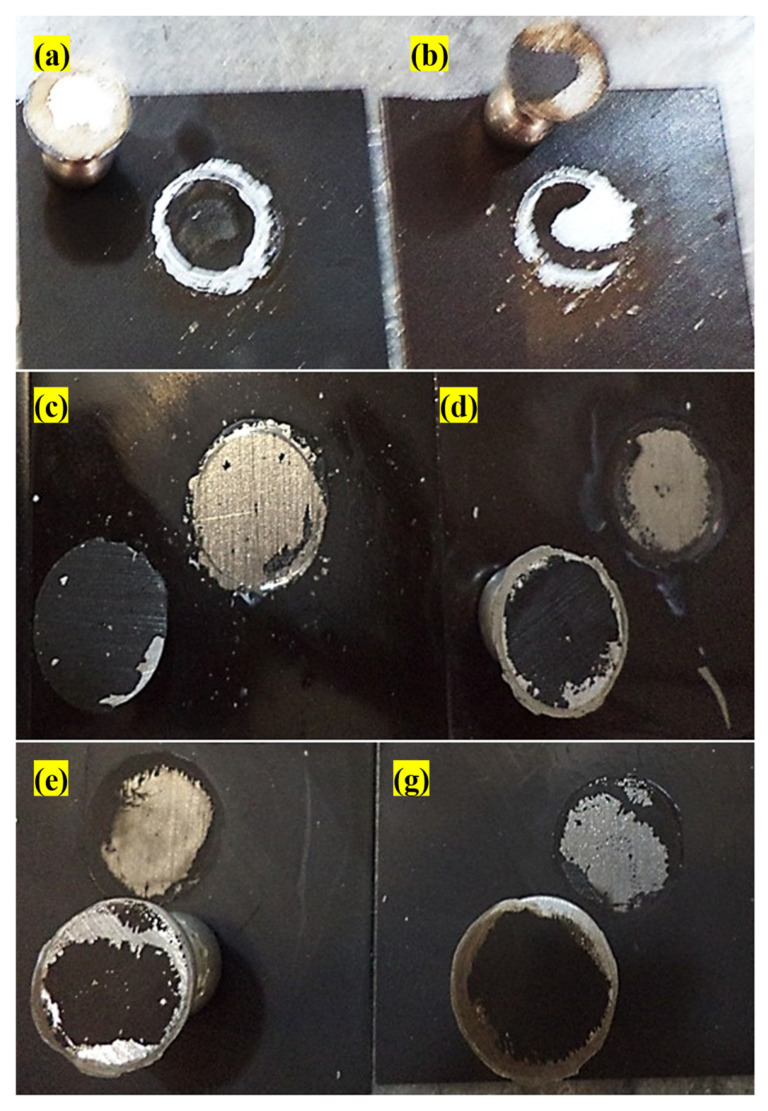
Photos of (**a**) 30 wt.% acrylic-based antifouling coating, (**b**) 40 wt.% acrylic-based antifouling coating, (**c**) 40 wt.% epoxy-based antifouling coating, (**d**) 60 wt.% epoxy-based antifouling coating, (**e**) 40 wt.% vinyl/rosin-based antifouling coating, (**g**) 50 wt.% vinyl/rosin-based antifouling coating after pull-off test.

In this test, all affecting factors were considered to be the same to compare the adhesion of the samples. The results of this test are reported in MPa based on the force required to separate the pin (dolly) attached to the coating from the substrate. Table 7 lists the results of the paint adhesion test of the prepared antifouling paints at different biocide contents. Figure 6 displays the photo of the pins and samples after running the tests. Acrylic resins had good adhesion to their substrate due to the polarity of the functional groups in the structure of the resins.

On the other hand, the adhesion of epoxy and vinyl/rosin coatings was much higher than that of the acrylic resin. The reasons for this were higher chemical and surface absorption and mechanical interlocking. The high polarity of the epoxy resin chain network, the presence of hydroxyl and ether aliphatic groups, and the presence of metal oxide on the steel surface caused electromagnetic absorption between the two materials. The adhesion of epoxy resin paints is related to their hydroxyl group [92]. The formation of chemical bonds between active hydrogen on the steel surface and the epoxide group distributed in the resin causes adhesion. In addition, the polymerization of the epoxy resin occurs after adding hardener and coating on the steel surface; performing this reaction in the cavities and pores created in the sample preparation increases the mechanical adhesion.

### 3.8. Gloss Test

The outcomes of the gloss test for prepared resins with diverse biocide contents are summarized in Table 8. The larger the gloss number, the more gloss the sample has. The gloss decreased as the biocide content increased, and the paint became dull. The reason is that the surface roughness increased with increased biocide content. As a result, when light radiation struck the surface, the surface reflected less radiation, scattering occurred, and the gloss decreased. In vinyl/rosin resin, due to the high oil absorption by rosin, which caused better miscibility of the biocides with the resin, it had less roughness and more gloss.

### 3.9. Impact Resistance Test

**Figure 7:**
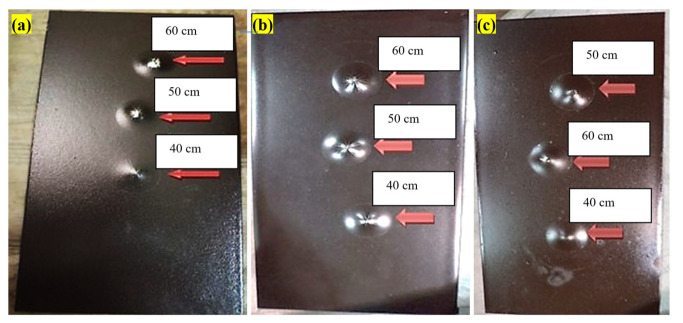
Photos of (**a**) acrylic-based antifouling coating, (**b**) epoxy-based antifouling coating, (**c**) vinyl/rosin-based antifouling coating after impact resistance test from different heights.

The photos of the samples after running the tests at each optimal content of biocides are shown in Figure 7. Tests were carried out 3 times for each sample. In all samples, the paint cracked after the ball was released from a height of 60 cm. In vinyl/rosin paint, the number of cracks was smaller compared with the other prepared samples due to the presence of rosin, and after the ball was thrown from a height of 50 cm, no cracks were seen. Due to its oily nature, rosin could reduce the tensile stress applied to the paint at the tip of the ball and increase the impact resistance. Epoxy and acrylic samples had lower impact resistance.

### 3.10. Abrasion Resistance Test

**Figure 8:**
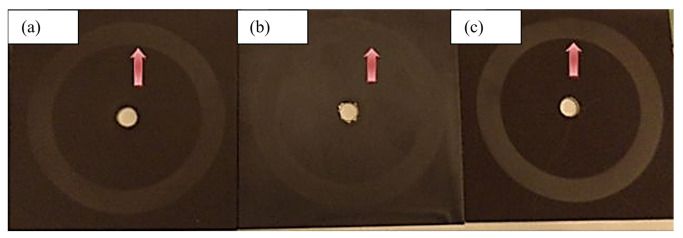
Photos of (**a**) acrylic-based antifouling coating, (**b**) epoxy-based antifouling coating, (**c**) vinyl/rosin-based antifouling coating after abrasion resistance test.

Since these tests are time-consuming, abrasion resistance tests were performed on only three samples of antifouling paint with optimal content of biocides to control fouling, and the results are given in Table 9 and Figure 8 shows the samples after the tests. The mass losses of acrylic resin, epoxy resin, and vinyl/rosin resin were 0.017, 0.016, and 0.001, respectively. As is obvious, most of the wear was seen in the acrylic sample because, in this resin, weak longitudinal bonds between monomeric chains caused the paint to have less resistance to abrasion and shear forces, but in epoxy and vinyl resins, bonds are established in all directions.

### 3.11. Scratch Resistance Test

Table 10 reports the force applied to the samples that caused the first scratch on the paint (g). According to the results, the lowest force required in order to create scratches was for acrylic antifouling paint with 30 wt.% content of biocides due to their lower modulus of elasticity. Moreover, the lower biocide content in acrylic-based paint reduced scratch resistance, since biocides can act as fillers and increase scratch resistance in paint. Biocides increase scratch resistance due to the hardness of resin, which has a lower elastic modulus. The highest scratch resistance was related to the epoxy resin, in which no scratch was seen under the weight of 3000 g because the occupation of intermolecular space between polymer chains by more biocides caused the stress applied by the needle of the device via hydrodynamic changes to be distributed between the polymer chains, and as a result, the paint resisted scratching.

### 3.12. Polarization TEST

**Figure 9:**
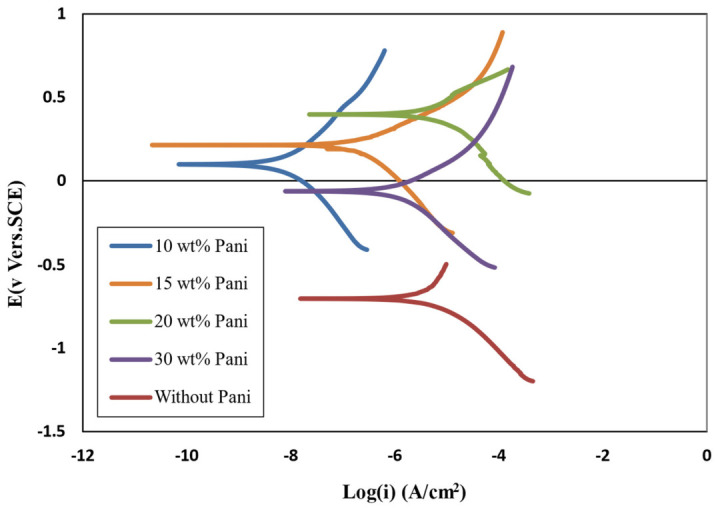
Polarization curves of different antifouling paints and sample with primer coating in 3.5% NaCl solution.

Figure 9 shows the polarization curves of different antifouling paints and the primer coating. The corrosion current density, potential, and corrosion rate of the samples are listed in Table 11. The results of the prepared resins were compared with a sample having only one primer coating to assess the effect of the antifouling paints. According to the results, it can be seen that the corrosion rate decreased, and the potential went toward positive values. The corrosion rate of the three paints was close to each other since their anticorrosion mechanism was the same. All three resins protected the substrate against corrosion by providing the barrier layer with high adhesion. Since the motion of ions in the interface between the metal and the coating depends on the amount of gravitational force between the metal and the coating, a stronger interaction between the coating and the metal and the presence of more gravitational forces in the interface increases the bond strength of the interface. This reduces the movement of ions in this area. In other words, the high adhesion of the coating to the metal surface causes the coating to act as an electrical insulator to prevent the formation of anode–cathode microcells on the metal surface or to minimize their formation.

### 3.13. Salt Spray Test

**Figure 10:**
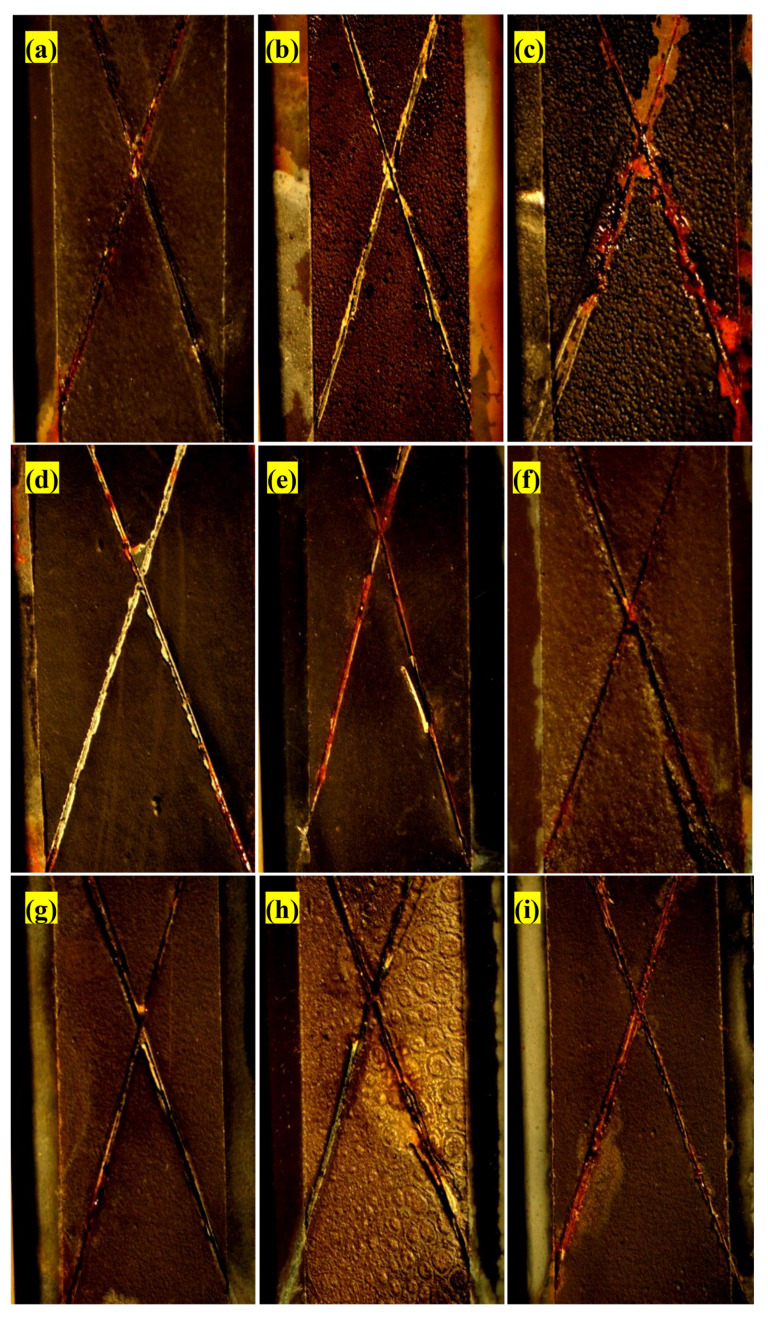
Corrosion impact after salt spray test of sample (**a**) 30 wt.% acrylic-based antifouling coating, (**b**) 40 wt.% acrylic-based antifouling coating, (**c**) 60 wt.% acrylic-based antifouling coating, (**d**) 20 wt.% epoxy-based antifouling coating, (**e**) 40 wt.% epoxy-based antifouling coating, (**f**) 60 wt.% epoxy-based antifouling coating, (**g**) 20 wt.% vinyl/rosin-based antifouling coating, (**h**) 40 wt.% vinyl/rosin-based antifouling coating, (**i**) 50 wt.% vinyl/rosin-based antifouling coating.

Figure 10 shows the impact of corrosion on the surface of the samples after salt spray tests. According to Figure 10, corrosion products can be seen in all samples in the scratch zone where the metal was exposed to a corrosive environment. The amount of corrosion in each sample depends on the resin’s nature and the biocides’ content. With the 40 wt.% acrylic-based antifouling coating, due to the incomplete distribution of pigments in the paint, non-uniformity occurred in the coating, which reduced the corrosion resistance. With the 60 wt.% acrylic-based antifouling coating, a large amount of corrosion products were formed due to small cracks in the paint. In general, the corrosion resistance of all the prepared paints was good. However, since they were denser than the acrylic-based paints, the epoxy- and vinyl/rosin-based paints had better anticorrosion performance than the acrylic ones. Furthermore, increasing the content of biocides reduced the corrosion resistance due to the increase in surface roughness.

The criterion for destroying organic coatings is swelling and lifting of the paint layer from the scratch area. The rate of adhesion loss of the coating layer increases with time and the penetration of corrosive agents into the coating layer. Paint layers applied to the steel surface can protect the surface from corrosion by forming a layer. A layer of paint on the surface containing zinc limits the activity of corrosive agents on the surface. For example, this layer prevents oxygen and water from entering the steel surface, stopping the cathodic reaction, which will reduce the corrosion rate. The most common failure mechanism of epoxy coatings against corrosion involves the gradual loss of adhesion of the coating to the steel substrate. In fact, due to the presence of scratches in the coating, the resin cannot completely prevent corrosive agents from reaching the metal surface. The interface resistance determines the corrosion resistance after corrosive agents reach the metal surface. The important thing is to maintain corrosion resistance over time. In fact, it is important to maintain adhesion over time for coatings used in corrosive environments. However, due to environmental conditions and mechanical and chemical damage, these coatings cannot provide complete protection over time.

In some cases, the damaged surface performs worse than a surface without coating. Through the interfacial layer of steel and paint, water can penetrate through the scratches. Water molecules form new hydrogen bonds with the hydrated surface of the metal, replacing the resin’s bonds with the metal, reducing adhesion, and thus detaching the paint coating. An anodic reaction at the site of the coating defect is usually accompanied by a cathodic reaction near the coating substrate, which results in further separation of the coating from the metal and, ultimately, more corrosion.

### 3.14. Investigation the Antifouling Property of Polyaniline in Paint and Its Efficacy on Corrosion Rate

**Figure 11:**
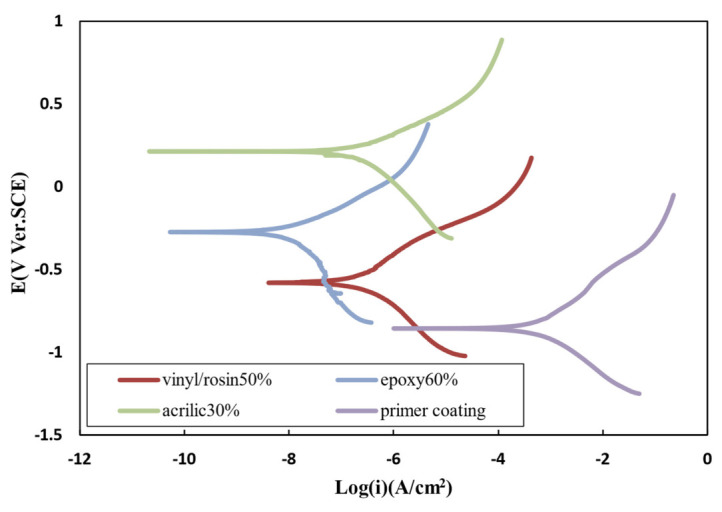
Polarization curves of antifouling paints at different contents of polyaniline in 3.5% NaCl solution.

Polyaniline was added to the epoxy-based paint after it was dissolved in dimethylformamide as an additive in proportions of 10, 15, 20, or 30 wt.%. Then, its effect on electrochemical properties was evaluated via Tafel analysis and electrochemical impedance, and its antifouling property was investigated. Epoxy-based antifouling paints containing 30 wt.% of biocide and 10, 15, 20, or 30 wt.% of polyaniline/acid were prepared and applied to steel. Then, a polarization test was conducted in a 3.5% NaCl solution. Polarization curves and data are shown in Figure 11 and Table 12, respectively. By comparing 4 paints containing polyaniline with a paint without polyaniline, it can be concluded that the corrosion potential was more positive, and the corrosion current density was significantly reduced with the addition of polyaniline. The corrosion potential increased by 0.75, 0.85, and 0.93 for paints containing 10, 15, and 20 wt.% polyaniline, respectively.

It should be noted that metals with coatings containing polyaniline are placed in the passive zone because a dense oxide layer is formed on the metal and polyaniline joint, protecting the metal against corrosion. An electric field is formed in the direction opposite to the electron transfer between the metal surface and the conductive coating surface (containing polyaniline), which can act as a charge transfer barrier layer to prevent the transfer of electrons from the metal to the oxidant and thus reduce corrosion. The corrosion potential decreased in the 30 wt.% sample, and the corrosion current density increased. The reason for this phenomenon is that at low concentrations of polyaniline (less than 20 wt.%), the distance between the conducting particles of polyaniline is large and fewer components are in contact, resulting in the formation of a chain of polyaniline, which can create charge conduction paths which reduce the strength of the coating. However, the conduction pathways are completed at higher concentrations, and adding polyaniline does not contribute to forming new conduction pathways in the paint. Even acid mixed with excess polyaniline may block the conduction channels in the paint.

**Figure 12:**
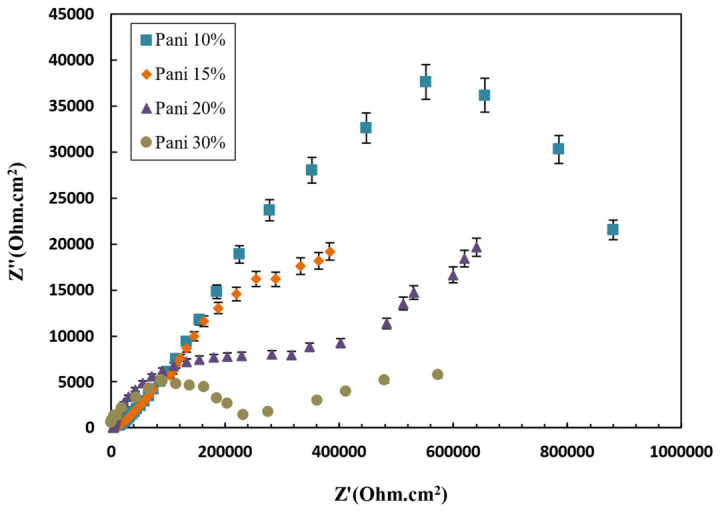
Impedance curves of antifouling paints at different contents of polyaniline in 3.5% NaCl solution.

Moreover, we observed that with an excessive increase of polyaniline, surface roughness increased. Density decreased, which increased surface defects, and the corrosive medium’s penetration rate into the paint coating was faster than the passive layer’s formation rate. As a result, the passive layer was destroyed by the corrosive medium before formation; thus, the anticorrosion effect of the coating was reduced. The impedance criterion is an important parameter for investigating the corrosion resistance behavior of polyaniline-containing coatings, which is directly related to the degree of penetration of the corrosive medium into the coating. Figure 12 shows the electrochemical impedance spectroscopy (EIS) results of antifouling polyaniline paints. As can be seen in Figure 12, the high-frequency curves are circular. The low-frequency curves are straight lines, which correspond to the Warburg region and indicate that the presence of polyaniline in the coating had a high protective effect and corrosion occurred only at the metal/environment interface, which was caused by the penetration of the corrosive medium into the pores [93]. Hence, the corrosion process largely depended on the mass transfer phenomenon and the electrochemical reaction. The sample curve of 10 wt.% polyanilines is only a semicircle with the highest impedance compared to other samples. In this sample, the process was often controlled by an electrochemical reaction, and the conductive coating effectively prevented the corrosive medium from penetrating the metal’s surface; this is also consistent with the polarization diagram. As the concentration of polyaniline and the surface roughness increased, and the density of the coating decreased, water penetrated the metal–coating interface, and corrosion began.

Since polyaniline is conductive, when paint containing polyaniline is applied to a layer of insulating paint on a metal substrate, the antifouling paint layer containing polyaniline plays the role of the anode, and the electrolysis of seawater produces Hyspochlorous acid (HOCl). HOCl covers the surface of the paint coating and prevents the formation and adhesion of marine organisms.

**Figure 13:**
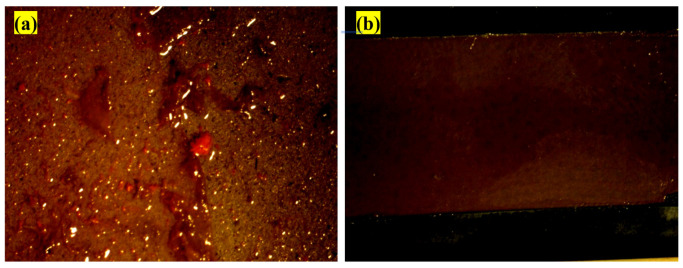
Photos of sample (**a**) without polyaniline, (**b**) containing 10 wt.% polyaniline after 3 months.

In traditional conductive coatings, which consist of the distribution of additives such as graphite, carbon black, and metal powders in organic or inorganic resins, the additive consumption is high, and it is unevenly distributed in the resin, so these coatings are very expensive, and their conductivity properties are not efficient. However, developing conductive polymers such as polyaniline, polythiophene, and pyrrole and mixing them with an acid improves conductivity. However, among these, polyaniline has the best miscibility with resin after mixing with acid. Therefore, a sample containing 10 wt.% of polyaniline was placed in an algae growth system for 3 months to evaluate its antifouling property, and Figure 13 shows photos of the samples after the test.

## 4. Environmental Challenges and Toxicity Effects of Biocides

Biocides are chemical substances that control or eliminate harmful organisms such as bacteria, viruses, fungi, and algae. While these compounds play a vital role in safeguarding public health and preventing the spread of diseases, their use poses significant environmental challenges and toxic effects. This summary will delve into two major aspects of these concerns: environmental impacts and the potential toxicity of biocides. The utilization of biocides can lead to a range of environmental challenges. One major issue is the persistence of these substances in ecosystems. Biocides, particularly those with high chemical stability, can accumulate in the environment over time, resulting in long-term contamination. This persistence poses risks to various organisms within the ecosystem, including aquatic organisms, birds, and mammals. Furthermore, biocides can undergo bioaccumulation, where they accumulate in the tissues of organisms at higher trophic levels. This bioaccumulation can magnify toxic effects, potentially disrupting entire food chains and ecological balances [94,95,96].

Another significant environmental challenge associated with biocides is the potential for non-target effects. Biocides are designed to target specific organisms, but their application may also affect non-target species, including beneficial insects, pollinators, and other organisms critical to ecosystem functioning. These unintended consequences can disrupt ecological interactions, reduce biodiversity, and impact ecosystems’ overall health and resilience. Additionally, the release of biocides into the environment via different pathways, such as runoff or leaching, can result in contamination of water, soil, and air, further exacerbating environmental challenges. In terms of toxicity, biocides have the potential to cause adverse effects on both human health and the environment. Some biocides are classified as hazardous substances due to their toxic properties. These substances may pose acute or chronic health risks to humans, ranging from skin and eye irritation to respiratory problems and even carcinogenic effects. Moreover, exposure to biocides has been linked to adverse effects on aquatic organisms, such as fish and amphibians, as well as on soil organisms and beneficial microorganisms critical for soil fertility and nutrient cycling.

In conclusion, the use of biocides presents significant environmental challenges and toxic effects. The persistence of these substances in ecosystems, potential non-target effects, and the toxicity risks they pose to both human health and the environment underscores the need for responsible and judicious use of biocides. Implementing appropriate risk assessment and management strategies, exploring alternative pest control methods, and promoting sustainable practices can help mitigate the negative impacts associated with biocides while ensuring effective disease prevention and control.

## 5. Conclusions

This study investigated the critical role of biocide release rates in the efficiency of antifouling paints. It examined the release rates of Cu_2_O and ZnO biocides in paints with varying resin compositions, particle sizes, and immersion times. Acrylic resin-based paints exhibited higher release rates due to the hydrolysis of acrylic copolymers, which weakened bonds and facilitated biocide release. Conversely, epoxy resin-based paints had lower release rates due to their insolubility and high crosslinking density, limiting water penetration and biocide dissolution. Vinyl/rosin-based paints showed intermediate release rates due to the combined hydrophobic properties of their constituent resins. Immersion time was found to be significant, with initial high release rates followed by a steady state, attributed to the formation of penetration barriers and biocide depletion. Biocide content in the formulation influenced release rates, showing a linear increase until a threshold was reached. Particle size also played a role, with smaller particles having higher release rates, especially in epoxy and vinyl/rosin resins. Interestingly, ZnO biocides exhibited release rates comparable to those of Cu_2_O despite their lower concentration. The study emphasized the importance of maintaining optimal release rates for effective antifouling performance, with fouling growth observed in samples with rates below recommendations. Curing time and hardness varied among different resin types while meeting standard requirements for physical properties.

## Figures and Tables

**Table 1:** Chemical composition of St-37 steel.

Element	Mn	P	S	C	Fe
Percent (wt.%)	0.6	0.03	0.02	0.17	0.18

**Table 2:** Composition of Cu_2_O and ZnO biocide paints at different contents and the role of used additives.

Material	Role	wt.%
Resin (binder)	Film-forming component of paint	10, 20, 30, 40, 50, 60, and 70 ± 0.1
Cu_2_O particles	Biocide activity	0, 7, 14, 20, 27, 33, and 40 ± 0.1
ZnO particles	Biocide activity	0, 3, 6, 10, 13, 17, and 20 ± 0.1
Titanium dioxide (pigment)	Providing the color of the paint	5 ± 0.1
Iron (III) oxide red (pigment)	Providing the color of the paint	5 ± 0.1
Dioctyl phthalate	Providing better pigment dispersion	5 ± 0.1
Talc	Providing the color of the paint	5 ± 0.1
Efka FA 4644	Dispersing agent	2 ± 0.1
BYK310	Providing uniform dispersion	2 ± 0.1

**Table 3:** Release rates of Cu_2_O and ZnO biocides at different contents and 5-, 10-, and 15-day intervals.

Resin	Total Biocide Content (wt.%)	Cu_2_O Content in Paint (wt.%)	ZnO Content in Paint (wt.%)	$\mathrm{Release}\;\mathrm{Rate}\;(\frac{\mu g}{{cm}^{2}.day})$					
				5 Days		5–10 Days		10–15 Days	
				${Zn}^{+}$	${Cu}^{2+}$	${Zn}^{+}$	${Cu}^{2+}$	${Zn}^{+}$	${Cu}^{2+}$
Acrylic	0	0	0	0	0	0	0	0	0
	10	7	3	7	9	3	3	3	2
	20	14	6	20	19	10	9	11	9
	30	20	10	31	29	13	14	12	14
	40	27	13	49	41	18	15	22	23
	50	33	17	53	60	25	18	25	20
	60	40	20	70	64	29	21	27	24
Epoxy	0	0	0	0	0	0	0	0	0
	10	7	3	5	0	5	3	5	0
	20	14	6	12	7	10	4	8	5
	30	20	10	13	6	9	6	7	5
	40	27	13	18	10	9	9	9	7
	50	33	17	25	14	12	10	10	8
	60	40	20	34	20	15	11	16	12
Vinyl	0	0	0	0	0	0	0	0	0
	10	7	3	5	3	2	0	2	2
	20	14	6	10	7	4	2	4	0
	30	20	10	16	9	9	3	8	3
	40	27	13	23	14	12	8	10	10
	50	33	17	35	21	18	14	16	15
	60	40	20	47	26	23	16	26	15

**Table 4:** Mean release rates of Cu_2_O and ZnO biocides at optimal contents after 5 days.

Resin	Total Biocide Content (wt.%)	$\mathrm{Release}\;\mathrm{Rate}\;(\frac{\mu g}{{cm}^{2}.day})$	
		${Zn}^{+}$	${Cu}^{2+}$
Acrylic	30	31	29
Epoxy	60	34	20
Vinyl	50	35	21

**Table 5:** Release rates of Cu_2_O and ZnO biocides at different contents, particle sizes, and 5-, 10-, and 15-day intervals.

Resin	Total Biocide Content (wt.%)	Particle Size (µm)	$\mathrm{Release}\;\mathrm{Rate}\;(\frac{\mu g}{{cm}^{2}.day})$					
			5 Days		5–10 Days		10–15 Days	
			${Zn}^{+}$	${Cu}^{2+}$	${Zn}^{+}$	${Cu}^{2+}$	${Zn}^{+}$	${Cu}^{2+}$
Acrylic	20	20	9	11	9	10	19	20
		50	4	5	8	8	16	20
	40	20	23	22	15	18	41	49
		50	19	15	12	14	29	40
Epoxy	20	20	5	8	4	10	7	12
		50	2	5	2	4	5	5
	40	20	7	9	9	9	10	18
		50	3	4	3	3	4	8
Vinyl/rosin	20	20	0	4	2	4	7	10
		50	0	3	0	3	5	6
	40	20	10	10	8	12	14	23
		50	3	6	5	7	10	19

**Table 6:** The pencil hardness and König pendulum hardness results of prepared antifouling paints.

Resin	Total Biocide Content (wt.%)	Pencil Hardness Degree	König Pendulum Oscillation Time (s)
Acrylic	10	B	65
	30	H	67
	40	3H	70
Epoxy	20	5H	91
	40	5H	127
	60	6H	165
Vinyl/rosin	20	4H	78
	40	4H	109
	50	B	65

**Table 7:** Obtained dolly separation force at diverse biocides content of acrylic, epoxy, and vinyl/rosin resins.

Resin	Total Biocide Content (wt.%)	Pin (Dolly) Separation Force (MPa)
Acrylic	30	12/4
	40	61/4
Epoxy	40	58/9
	60	41/8
Vinyl/rosin	40	61/6
	50	17/7

**Table 8:** Gloss test results at diverse biocides content of acrylic, epoxy, and vinyl/rosin resins.

Resin	Total Biocide Content (wt.%)	Lux
Acrylic	30	26
	40	20
Epoxy	40	42
	60	40
Vinyl/rosin	40	45
	50	38

**Table 9:** Mass loss of the samples after abrasion resistance test.

Resin	Total Biocide Content (wt.%)	Initial Weight (g)	Final Weight (g)	Mass Loss (g)
Acrylic	30	62.847	62.83	0.017
Epoxy	60	64.196	64.18	0.016
Vinyl/rosin	50	63.301	63.30	0.001

**Table 10:** Required force for the scratch resistance of the prepared paints.

Resin	Total Biocide Content (wt.%)	Applied Weight (g)	Photo of Formed Scratch
Acrylic	30	1500	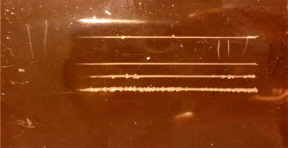
Epoxy	60	3000	No scratch
Vinyl/rosin	50	2000	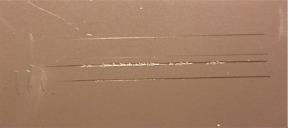

**Table 11:** Obtained data from the polarization curve of antifouling paints and primer coating.

Resin	E_corr_ (V)	$I_{\mathrm{corr}}\times 10^{-6}$ ($\frac{A}{{cm}^{2}}$)	$\mathrm{Corrosion}\;\mathrm{Rate}\;(\frac{mm}{year})$
Primer coating	−0.83	79	3.61
Acrylic 30 wt.%	−0.39	3.2	0.128
Epoxy 60 wt.%	−0.27	0.082	0.013
Vinyl/rosin 50 wt.%	−0.58	0.47	0.0188

**Table 12:** Obtained data from the polarization curve of antifouling paints containing polyaniline.

Polyaniline Concentration (wt.%)	E_corr_ (V)	$I_{\mathrm{corr}}\times 10^{-6}$ $\;(\frac{A}{{cm}^{2}})$	$\mathrm{Corrosion}\;\mathrm{Rate}\;(\frac{mm}{year})$
0	−0.73	6.3	0.24
10	0.11	0.0093	0.00035
15	0.23	0.37	0.0148
20	0.47	8.7	0.348
30	−0.07	3.1	0.11

## Data Availability

All data generated or analyzed during this study are included in this published article.

## Supplementary Materials

The following supporting information can be downloaded at: https://www.mdpi.com/article/10.3390/polym15193948/s1, Table S1: Specification of artificial seawater; Figure S1: Real photo of the designed laboratory system; Figure S2: Schematic of the designed laboratory system; Figure S3: Real photo of built setup for algae growth; Table S2: Chemical composition of used water in the algae growth environment.
