# Supplementary material for: Investigation of the Release Rate of Biocide and Corrosion Resistance of Vinyl-, Acrylic-, and Epoxy-Based Antifouling Paints on Steel in Marine Infrastructures
Source: Polymers (Basel). 2023 Sep 29;15(19):3948. doi: 10.3390/polym15193948 (PMC10575080; doi:10.3390/polym15193948)
Supplement: Supplementary file 1 [file polymers-15-03948-s001.zip › polymers-2623807-supplementary.pdf]

Investigation of the release rate of biocide and corrosion resistance of vinyl-, acrylic-, and epoxy-based antifouling paints on steel in marine infrastructures

Adel Jalaie[a], Abdolah Afshaar[a*], Seyed Borhan Mousavi[b**], Mohammad Heidari[c]

[a] Department of Materials Science and Engineering, Sharif University of Technology, Tehran, Iran

[b] J. Mike Walker ‘66 Mechanical Engineering Department, Texas A&M University, College Station, TX 77843, USA

[c] Faculty of Chemical & Petroleum Engineering, University of Tabriz, Tabriz, Iran

[*]Corresponding 1 email: abdolahafshaar@gmail.com

[**]Corresponding 2 emails: seyedborhanm@gmail.com, borhanm@tamu.edu

Table S1 Specification of artificial seawater.

Material	Concentration (gr/liter)
NaCl	24.53
$MgCl_2.6H_2O$	5.20
Na_2SO_4	4.09
$CaCl_2$	1.16
KCl	0.695
$NaHCO_3$	0.201
H_3BO_3	0.207

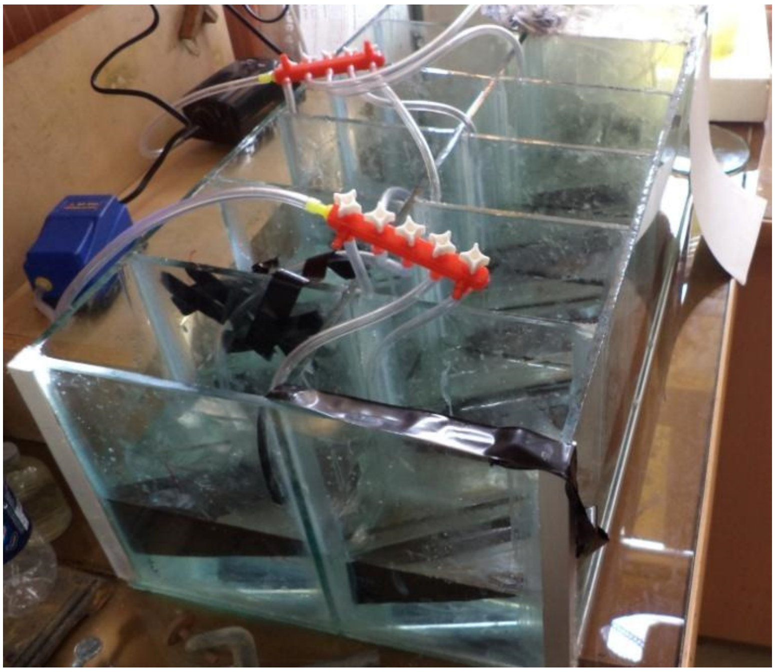

Figure S1. Real photo of the designed laboratory system.

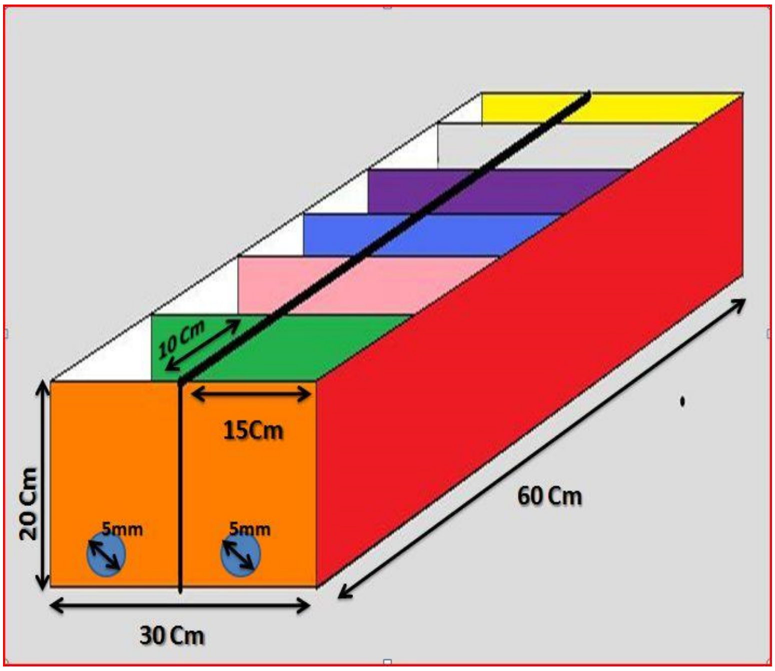

Figure S2. Schematic of the designed laboratory system.

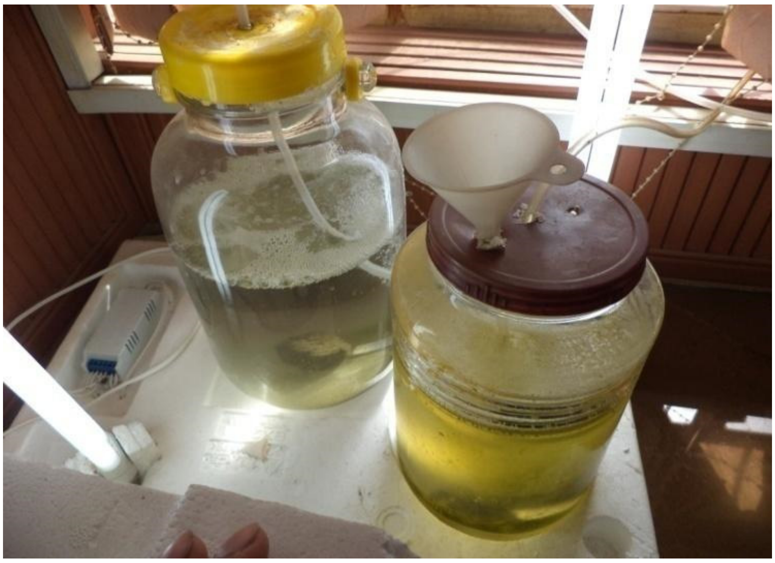

Figure S3. Real photo of built setup for algae growth.

Table S2 Chemical composition of used water in the algae growth environment.

Material	Concentration (gr/dm^3)
K_2HPO_4	2
KH_2PO_4	3.4
$MgSO_4.7H_2O$	2
$NaNO_3$	0.6
$CaCl_2.2H_2O$	0.6
NaCl	0.5
KOH	3
$FeSO_4.7H_2O$	0.5
H_2SO_4	0.184
H_3BO_3	1
$ZnSO_4.7H_2O$	0.3
$MnCl_2.4H_2O$	0.05
$CuSO_4.5H_2O$	0.06
$Co(NO_3)_2.6H_2O$	0.02
$Na_2MoO_4.2H_2O$	0.04
